# Development of the VISAGE enhanced tool and statistical models for epigenetic age estimation in blood, buccal cells and bones

**DOI:** 10.18632/aging.202783

**Published:** 2021-03-11

**Authors:** Anna Woźniak, Antonia Heidegger, Danuta Piniewska-Róg, Ewelina Pośpiech, Catarina Xavier, Aleksandra Pisarek, Ewa Kartasińska, Michał Boroń, Ana Freire-Aradas, Marta Wojtas, Maria de la Puente, Harald Niederstätter, Rafał Płoski, Magdalena Spólnicka, Manfred Kayser, Christopher Phillips, Walther Parson, Wojciech Branicki, VISAGE Consortium

**Affiliations:** 1 Central Forensic Laboratory of the Police, Warsaw, Poland; 2 Institute of Legal Medicine, Medical University of Innsbruck, Innsbruck, Austria; 3 Department of Forensic Medicine, Jagiellonian University Medical College, Krakow, Poland; 4 Malopolska Centre of Biotechnology, Jagiellonian University, Krakow, Poland; 5 Forensic Genetics Unit, Institute of Forensic Sciences, University of Santiago de Compostela, Santiago de Compostela, Spain; 6 Department Medical Genetics, Warsaw Medical University, Warsaw, Poland; 7 Department of Genetic Identification, Erasmus MC University Medical Center Rotterdam, Rotterdam, Netherlands; 8 Forensic Science Program, The Pennsylvania State University, University Park, PA 16802, USA

**Keywords:** DNA methylation, bisulfite amplicon MPS, epigenetic age prediction tool, age prediction in blood and buccal cells, age prediction in bones

## Abstract

DNA methylation is known as a biomarker for age with applications in forensics. Here we describe the VISAGE (VISible Attributes through GEnomics) Consortium’s enhanced tool for epigenetic age estimation in somatic tissues. The tool is based on eight DNA methylation markers (44 CpGs), bisulfite multiplex PCR followed by sequencing on the MiSeq FGx platform, and three statistical prediction models for blood, buccal cells and bones. The model for blood is based on six CpGs from *ELOVL2*, *MIR29B2CHG*, *KLF14*, *FHL2*, *TRIM59* and *PDE4C*, and predicts age with a mean absolute error (MAE) of 3.2 years, while the model for buccal cells includes five CpGs from *PDE4C*, *MIR29B2CHG*, *ELOVL2*, *KLF14* and *EDARADD* and predicts age with MAE of 3.7 years, and the model for bones has six CpGs from *ELOVL2, KLF14, PDE4C* and *ASPA* and predicts age with MAE of 3.4 years. The VISAGE enhanced tool for age estimation in somatic tissues enables reliable collection of DNA methylation data from small amounts of DNA using a sensitive multiplex MPS assay that provides accurate estimation of age in blood, buccal swabs, and bones using the statistical model tailored to each tissue.

## INTRODUCTION

The use of epigenome-wide association studies has provided a deeper insight into individual differences and fluctuations in DNA methylation levels in the human genome. Many studies have identified age-related differentially methylated regions (DMRs) and sites (DMSs) that have the potential to predict epigenetic age in various human tissues [e.g. 1–4]. These studies indicated that epigenetic age is highly correlated with chronological age but have also revealed differences in epigenetic aging rates amongst individuals. The epigenetic aging rate was further correlated with lifespan, as well as age-related traits and diseases [5–8]. Although the potential of epigenetic age prediction in biomedical sciences needs further investigation, one promising application is found in forensics.

DNA-based age prediction in forensic applications can be used for intelligence purposes to gain information from unknown individuals who have left their DNA at a crime scene or whose remains are subjected to genetic identification. Perpetrators, who remain unknown to the investigating authorities from their forensic DNA profiles, cannot be identified with standard DNA profiling techniques. In recent years, forensic DNA phenotyping has emerged as an approach that can obtain information of an unknown crime scene sample donor on their appearance and bio-geographic ancestry from crime scene DNA [9]. This allows for focused police investigations to help characterize unknown perpetrators, where estimating age from crime scene DNA would also be highly informative. Age is an important phenotypic feature that manifests itself through a set of visible attributes that are difficult to hide or modify, and therefore can be very effective in narrowing down the number of potential suspects in the context of forensic DNA phenotyping. In particular, aging is reflected in several features of human visible characteristics like hair greying, hair loss, facial wrinkles and other signs of aging skin [10]. Thus, reliable DNA-based prediction of appearance traits as a forensic intelligence tool is ideally accompanied with age estimation.

A number of methods that use DNA methylation markers to predict age have been reported. Such methods can be classified into two main categories according to the number of CpG sites included. Large scale methods incorporate hundreds of loci [2, 3, 5, 11] and require DNA microarray technology to collect the data necessary for using predictive algorithms. Since forensic genetics is very demanding in terms of the sensitivity of the methods applied, smaller sets of markers that can be analyzed using lower DNA input methods are more suitable. Most of the epigenetic age prediction methods proposed in the forensic field have been designed to predict age in blood. Weidner et al. (2014) developed a 3-CpG model involving genes *ASPA*, *ITGA2B* and *PDE4C*, and using pyrosequencing technology that allowed age estimation with a mean absolute error (MAE) of 4.12 years. Several smaller age predictive tests were then proposed in forensics [12, 13]. The test developed by Zbieć-Piekarska offered a set of markers suitable to predict age from blood in Europeans and Asians [14]. The method involved analysis of five DMSs in *ELOVL2*, *TRIM59*, *C1orf132/MIR29B2CHG*, *KLF14* and *FHL2*, that provided prediction accuracy in blood with a MAE of 3.7 and 4.2 years in Polish and Korean populations, respectively [12, 14]. Importantly, these markers also showed similar accuracy for age prediction in saliva (MAE = 3.6 years) [15] and buccal swabs (MAE = 4.3 years) [15]. Moreover, some of these markers also showed a correlation with age in bones and teeth [16–18]. Blood (especially bloodstains), buccal swabs, and skeletal remains are commonly analyzed in forensic laboratories for human identification. Age estimation methods developed for these tissues may provide additional information to assist with the identification process. The VISAGE consortium has implemented these five markers in a basic tool for sensitive multiplex PCR amplification of bisulfite converted DNA followed by a massively parallel sequencing (MPS) on a MiSeq FGx instrument [19]. Indeed, due to the quantitative character of DNA methylation variation and the well-known method-to-method bias of DNA methylation analysis, predictive models based on data generated with one method, including public datasets based on DNA methylation microarrays [12, 15] cannot be easily adopted to interpret DNA methylation data generated with another method. Methylation analysis methods widely vary in forensic use and have hampered comparisons of the efficiency of qPCR [20], SNaPshot [21] and MPS [22].

MPS offers a universal solution to DNA variation analysis that can be applied to study DMSs as well as the established variation of single nucleotide polymorphism (SNPs) and short tandem repeats (STRs) [23]. Differences exist between MPS analysis of both marker types since the completely quantitative nature of DMS analysis contrasts with the mainly qualitative nature of SNP and STR analysis, so the MPS multiplex capacity is markedly lower for DMS analysis compared to SNP and STR analysis. Genotyping of bisulfite-converted DNA has become the standard for DNA methylation analysis. Although the design of targeted PCR-based MPS tests is difficult to apply to bisulfite-converted DNA, small scale multiplexing is possible and this could be a solution to the problem of measuring DNA methylation in forensic genetic tests, offering the right balance between sensitivity, throughput and reliability. The forensic community has made the first steps towards implementation of MPS for DNA methylation analysis [24–26].

In this study, we have advanced the development of epigenetic age estimation in forensics and present the VISAGE enhanced tool for age estimation of DNA from somatic tissues, combining eight age-informative DNA methylation markers into a bisulfite multiplex PCR for simultaneous targeted MPS and three new statistical models to predict age in blood, buccal cells and bones.

## RESULTS

### Assay development and optimization

Eight age-informative DNA methylation markers containing 44 CpG sites (Supplementary Table 1) were selected from the literature based on their reported age correlation in different somatic tissues such as blood, buccal cells and bones [11, 12, 27–31], and were successfully combined into one multiplex PCR assay for bisulfite-converted DNA. Five of these selected markers were included in the previously described VISAGE basic tool for estimating age from blood [19]. Primer pairs targeting three newly selected markers, *EDARADD*, *PDE4C* and *ASPA* were added sequentially to the multiplex assay of the VISAGE basic age tool [19] to monitor the effect of each added marker individually and to finally achieve a functioning multiplex for all eight markers (Table 1).

**Table 1:** Primer sequences and concentrations of the final PCR multiplex.

**Marker**	**Primer sequence (5'-3')**	**CpGs [N]**	**Amplicon size [bp]**	**Amp. position (GRCh38)**	**Strand**	**Final conc. [μM]**	**Design**
*FHL2*	fwd: TGTTTTTAGGGTTTTGGGAGTATAG	1	167	Chr2:105399250-105399416	+	0.2	[12]
	rev: ACACCTCCTAAAACTTCTCCAATCTCC	1					
*KLF14*	fwd: GGTTTTAGGTTAAGTTATGTTTAATAGT	1	128	Chr7:130734307-130734434	+	0.2	[12] ^a^
	rev: ACTACTACAACCCAAAAATTCC	0					
*TRIM59*	fwd: TATAGGTGGTTTGGGGGAGAG	1	141	Chr3:160450140-160450280	+	0.2	[12]
	rev: AAAAAACACTACCCTCCACAACATAAC	1					
*ELOVL2*	fwd: AGGGGAGTAGGGTAAGTGAG	1	267	Chr6:11044500-11044766	+	0.2	[28]
	rev: AAACCCAACTATAAACAAAACCAA	0					
*MIR29B2CHG*	fwd: GTAAATATATAAGTGGGGGAAGAAGGG	1	146	Chr1:207823605-207823750	+	0.4	[12]
	rev: TTAATAAAACCAAATTCTAAAACATTC	0					
*EDARADD*	fwd:TTGGTGATTAGGAGTTTTAGTGTTTT	0	193	Chr1:236394309-236394501	-	0.4	[28]
	rev: CCACCTACAAATTCCCCAAA	0					
*ASPA*	fwd: TTTTGGAGGAATTTATGGGAA	0	108	Chr17:3476207-3476314	+	0.4	present study
	rev: ATAAATAATTTTACCTCCAACCCTA	0					
*PDE4C*	fwd: TTGTAGGAGGAAAAGGGTTAG	1	215	Chr19:18232953-18233167	+	0.4 or 1^b^	present study
	rev: AAAACAAAAACTTACAACAAATTAAA	0					

^a^forward primer was adapted to match GRCh38.

^b^assay design 1: 0.4 μM; assay design 2: 1 μM.

**Figure 1:**
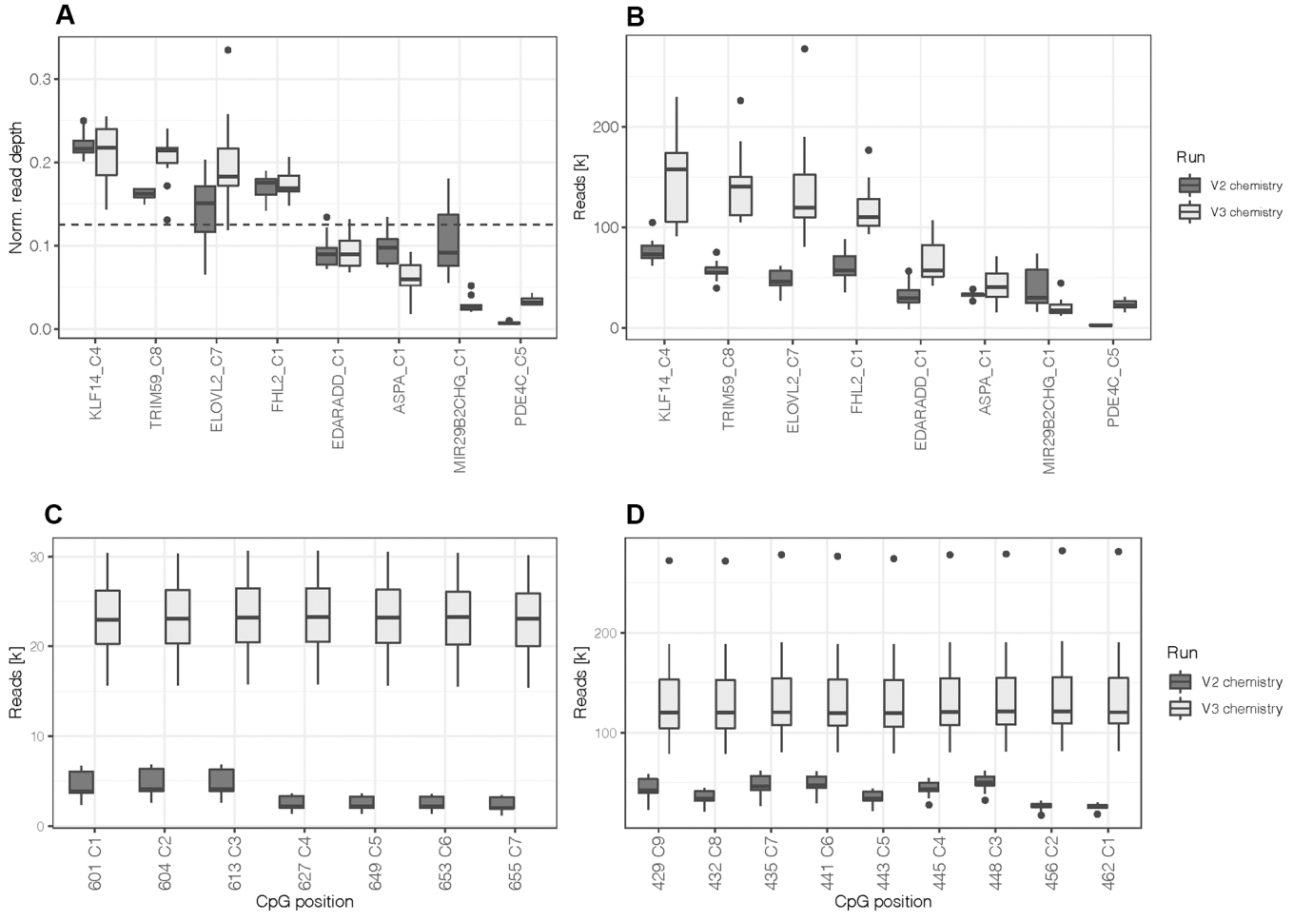
(**A**) Normalized read depth was calculated by selecting one CpG site per marker to assess for read distribution between amplicons. The dashed line indicates the expected value per marker (0.125) in case of a perfectly balanced distribution. (**B**) Read depth at one CpG site per marker. (**C**) Read depth at all CpGs located in the target sequence of *PDE4C* and (**D**) *ELOVL2*. All boxplots compare DNA methylation standards processed with the first assay design using the MiSeq reagent kit v2 (N = 10) and the re-optimized assay (design 2) using the MiSeq reagent kit v3 (N = 12).

The design was first tested with artificially methylated DNA standards (N = 10) at an optimum input DNA amount for bisulfite conversion of 200 ng using the MiSeq Reagent Kit v2. Sequence read depth at all 44 CpGs covered by the primer design of the eight DNA methylation markers exceeded the minimum of 1,000 reads (mean = 42,012.1 ± 21,282.7 paired reads) set for accurate methylation quantification [32]. Measured methylation values of the differentially methylated DNA standards showed robust quantification with an average difference between duplicates of 1.3% ± 2.1% (one CpG per marker; Supplementary Figure 1). As expected from the lower PCR product yields for *PDE4C,* CpG positions located in the target sequence of this marker produced low read depths (mean = 3,386.3 ± 1,630.4 paired reads) compared to the other markers. Figure 1A illustrates normalized read depth of one CpG site per marker, clearly indicating the lower sequence output of *PDE4C* C5. Furthermore, the read depths of the CpGs located in *PDE4C* (7 CpGs) and *ELOVL2* (9 CpGs) were lower for positions that were not covered during sequencing from both ends (Figure 1C, 1D). This results from the read length of the used sequencing kit (2 x 150 cycles), which is not sufficient to cover the whole targeted region of the two longest amplicons from both directions (*ELOVL2*: 267 bp and *PDE4C*: 215 bp).

Aiming at a balanced PCR multiplex, the concentrations of the *PDE4C* primers were increased equimolarly and more balanced amplicon yields were obtained at an assay concentration of 1 μM (Supplementary Figure 2). The altered PCR multiplex was tested using the MiSeq Reagent Kit v3 and methylated DNA standards (N = 12). Read depth of *PDE4C* CpGs was increased to 23,005.7 ± 4,659.5 paired reads. When comparing normalized read depths to earlier generated data, the performance of *PDE4C* was enhanced, but a fully balanced sequence read distribution was not achieved. This was observed in the relative decrease of read depth at *MIR29B2CHG* (Figure 1A). However, on average the v3 kit led to an approximately doubled sample coverage (v3: 701,330.8 ± 147,289.9 versus v2: 348,679.2 ± 62,647.1 paired reads) and an increase in mean read depth of 45,109.4 paired reads (overall mean = 87,121.5 ± 54,854.7, Figure 1B). The number of reads at target CpG positions on *MIR29B2CHG* still yielded a mean of 20,2435 ± 8,741 paired reads. Additionally, the longer read length of the v3 kit enabled constant read depths at all the CpGs of *ELOVL2* and *PDE4C* across the targeted sequences (Figure 1C, 1D). The sequence quality control showed that all 44 target CpG positions had a misincorporation rate below 0.4% (mean = 0.04%) and the calculated bisulfite conversion efficiency exceeded 99.6% for all samples.

### Evaluation of MPS assay performance

**Figure 2:**
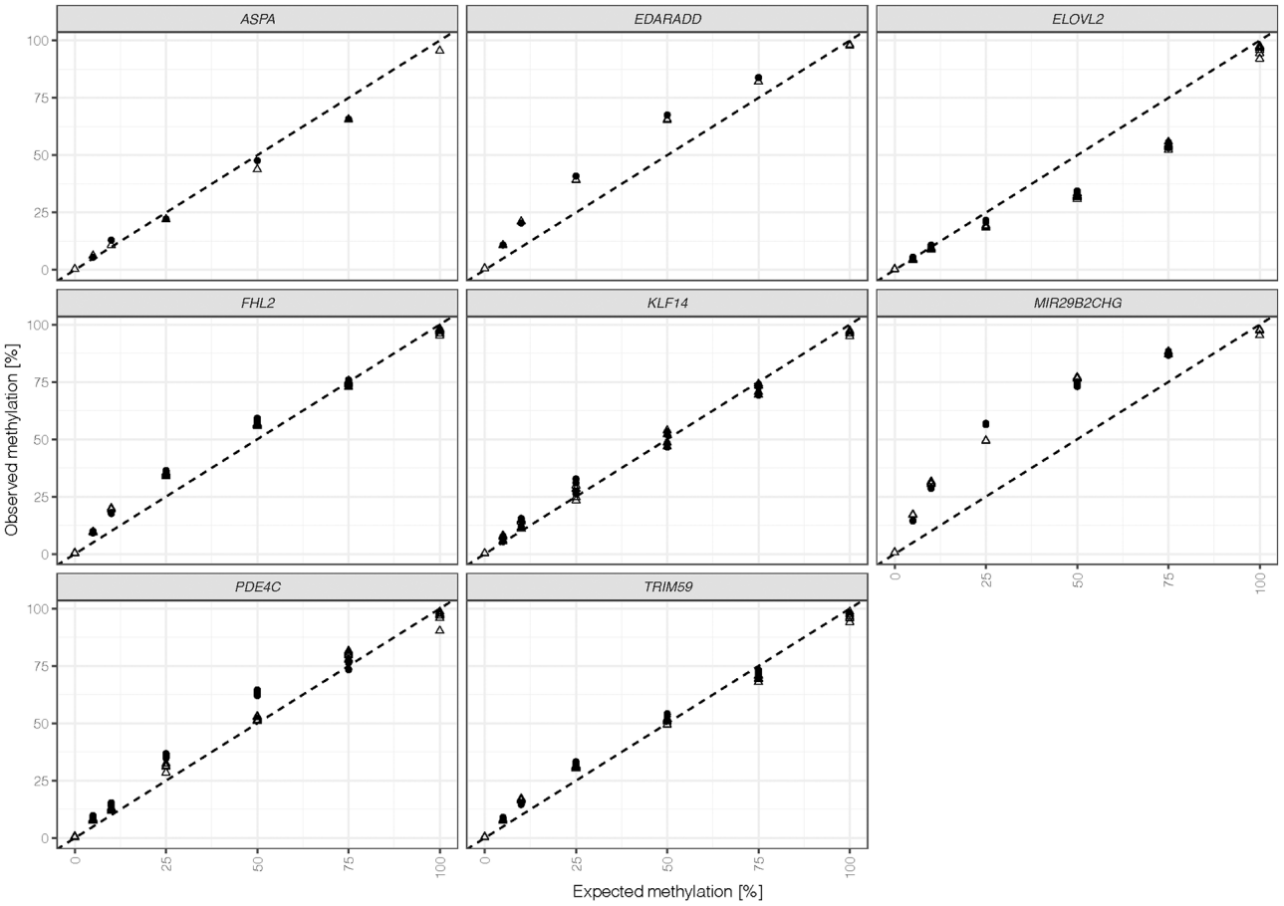
**Methylation quantifications obtained for duplicates (indicated by shape) were calculated for all 44 target CpG sites (ASPA: 1 CpG, *EDDARAD*: 2 CpGs, *ELOVL2*: 9 CpGs, *FHL2*: 10 CpGs, *KLF14*: 4 CpGs, *MIR29B2CHG*: 3 CpGs, *PDE4C*: 7 CpGs, *TRIM59*: 8 CpGs).** The dashed line represents the line of identity (intercept = 0, slope = 1).

Seven differentially methylated DNA standards were assessed with the final optimized assay using the v3 kit. The methylation quantification of all 44 CpGs versus the expected methylation is shown in Figure 2. Measured methylation levels at most CpGs were close to the line of identity, indicating good overall concordance between experimentally determined and actual methylation levels. CpG sites at *MIR29B2CHG* and *EDARADD* showed an overestimation of methylation levels, while CpGs at *ELOVL2* exhibited a bias towards unmethylated Cs. The methylation quantification obtained appeared to be robust with an average difference between duplicates of 1.9% ± 1.2% across markers and methylation levels ranging from 5% to 75% (Supplementary Table 2). However, two outliers were detected: both the 50% methylated sample at *PDE4C* positions and the 25% methylated sample at *MIR29B2CHG* showed higher variation (12% and 7.5%, respectively) between duplicates. Potential variability between the target CpG sites throughout the same amplicon was explored by calculating the maximum difference in methylation at one marker and sample (excluding *ASPA*, N = 1). Overall, these differences were low with a mean of 1.8% ± 2% across markers and ratios. The highest variation was detected at *KLF14* with 3.9% ± 2.4% when comparing amplicons, which at the same time showed the most stable methylation quantification (mean difference between duplicates = 0.8% ± 1%). Further evaluation of DNA standards at different methylation levels showed that the highest differences were observed at 100% expected methylation with a mean of 3.8% ± 2.6%.

Next, the 50% methylated DNA standard was used to test the sensitivity of the MPS assay with 200 ng, 100 ng, 50 ng, 20 ng, 10 ng and 1 ng input DNA in duplicate reactions. Although sample coverage decreased from 200 ng (mean = 374,254.5 paired reads) to 1 ng (mean = 280,105 paired reads), the lower input samples still showed high sequencing coverage values. One outlier at 10 ng was detected with lower coverage (76,464 paired reads) for one of the duplicates. This sample showed very low library quantification results (2.6 nM) in comparison to the median concentration of the sensitivity dilution series’ libraries (319.7 nM) and a read depth below the 1,000 reads threshold for the *MIR29B2CHG* amplicon. Except for one further sample at 1 ng DNA input for *PDE4C*, all other replicates showed read depths above the 1,000 reads threshold at the 44 targeted CpG sites. Furthermore, base misincorporation rates remained below 0.6% down to 10 ng DNA input and below 1.3% for 1 ng samples. Bisulfite conversion efficiency exceeded 99.4% for all samples.

**Figure 3:**
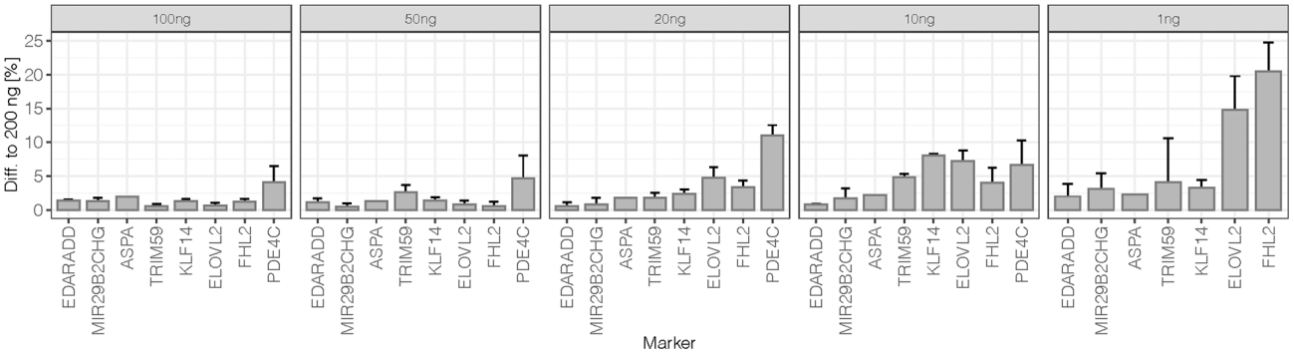
**Difference to 200 ng of sensitivity dilutions at 50% methylation level: The average difference per marker was calculated from mean obtained methylation values (N = 2) at all 44 target CpG sites.** The error bars represent the standard deviation at markers targeting more than one CpG (*EDARDD* 2 CpGs, *ELOVL2* 9 CpGs, *FHL2* 10 CpGs, *KLF14* 4 CpGs, *MIR29B2CHG* 3 CpGs, *PDE4C* 7 CpGs, *TRIM59* 8 CpGs). Due to the high difference of *PDE4C* at 1 ng (61.9%), the value is excluded from the plot.

Differences of mean methylation obtained for duplicates from 100 ng to 1 ng DNA input at all 44 CpGs were compared to the mean methylation level obtained for the 200 ng reference sample. The average differences and standard deviations per marker are shown in Figure 3. At 100 ng and 50 ng DNA inputs, methylation levels were close to those of the reference with 1.6% ± 1.1% and 1.7% ± 1.4% differences across the eight markers, respectively. Variability increased slightly from 50 ng to 20 ng (3.4% ± 3.9%) and from 20 ng to 10 ng (4.4% ± 2.7%). In particular, *PDE4C* showed higher variation at 10 ng with a mean difference of 11% to the optimum DNA input. A more detailed analysis of the 10 ng replicates showed an increased difference between duplicates (median = 6.0%) compared to the higher DNA input samples of the sensitivity study (median difference = 1.5% at 200 ng to 3.0% at 20 ng). Higher deviations from methylation values obtained for the reference DNA input were observed for the low quantity 10 ng sample (2.6 nM library) possibly introducing greater variation. Additionally, higher base misincorporation rates were observed within the *PDE4C* amplicon sequence of this duplicate (Supplementary Figure 3B). At 1 ng DNA input, methylation quantification became unreliable with extensive deviation from the values of the reference DNA input (14.0% ± 20.5%).

### Development of prediction model for blood, buccal cells and bones

**Figure 4:**
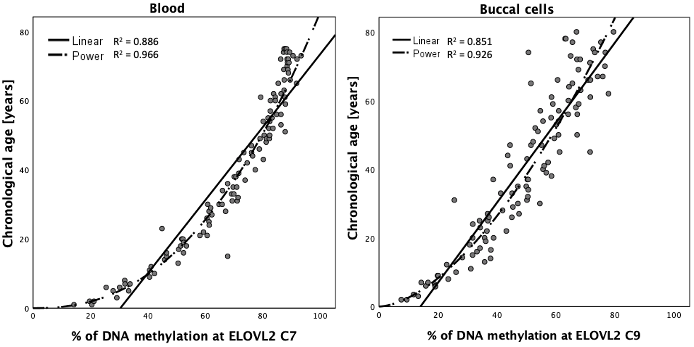
**Curve estimation for DNA methylation data at *ELOVL2* C7 in blood and *ELOVL2* C9 in buccal cells.**

DNA methylation data generated with the MPS tool in blood (N = 160), buccal swab (N = 160) and bone (N = 161) DNA samples were randomly divided into a training set (N = 112 each) and testing set (N = 48 blood and buccal cells and 49 bones) by retaining comparable distributions of age and sex between both sets as far as possible (Table 2). The correlation of DNAm in particular CpG sites with age in blood, buccal cells and bones was investigated in the appropriate training sets using univariate linear regression analysis. All 44 CpG sites at the eight DNA methylation markers covered by the MPS tool showed a statistically significant correlation with age in blood (Supplementary Table 1). For 26 CpG sites (59%) located in *ELOVL2*, *FHL2*, *TRIM59* and *PDE4C* very high β values were observed (>0.9) with single CpGs explaining R^2^ > 80% of the variation observed in age in blood samples (>90% for 8 CpG sites within *ELOVL2* and *FHL2*). The highest statistical significance was noted for *ELOVL2* C9 (β = 0.963; *P*-value = 9.724×10^-65^; R^2^ = 0.928). As with blood, all cytosines also showed significant correlation with age in buccal cells, but only in 13 of the sites (30%) were β values > 0.9 recorded. The highest significance was achieved for *PDE4C* C5 (β = 0.965; *P*-value = 3.648×10^-65^); by itself explaining R^2^ = 93.1% of the variation observed in age in buccal cells (Supplementary Table 1). In the case of bones, significant correlation with age was observed for all CpGs except C2 and C3 from *MIR29B2CHG*. The position C1 in *MIR29B2CHG* was weakly but significantly correlated with age (β = -0.24; *P*-value = 0.011). High significance and effect size with β > 0.8 were noted for *TRIM59* C3-C8 (*P*-value: 4.899×10^-34^; 1.699×10^-26^; 4.431×10^-27^; 5.537×10^-30^; 4.2×10^-32^; 8.743×10^-34^), *KLF14* C3 (*P*-value: 1.68×10^-33^), *ELOVL2* C2 (2.021×10^-33^), *FHL2* C5 (2.307×10^-26^) and C7 (6.591×10^-31^), as well as *PDE4C* C4 (2.492×10^-29^), C6 (6.886×10^-30^) and C7 (2.355×10^-26^). Since DNA methylation-age correlations may show non-linear patterns, various types of data transformation were also tested. Curve estimation analysis indicated a non-linear pattern for CpG sites within *ELOVL2* with power transformation best fitting the DNA methylation data for blood and buccal cells and thus power transformed data were used in modelling (Figure 4).

**Table 2:** Samples used in multivariable linear regression analysis and prediction modelling.

**DNA source**	**Sample number/sex**	**Mean age**	**Training set**			**Testing set**	
			**Sample number**	**Age range**		**Sample number**	**Age range**
Blood	160/80:80	40.2±22.7	112	1-75		48	1-75
Buccal cells	160/80:80	40.5±22.8	112^a^	2-80		48	2-80
Bones	161/129:32	46.1±14.8	112^a^	19-93		49^b^	22-75

^a^because of the missing data for *PDE4C* C5 or *ELOVL2* C2 one sample from the buccal cell training set and one sample from the bone training set were excluded and therefore the final number of samples to train the models was 111.

^b^three samples had missing data in *ELOVL2* C2.

**Figure 5:**
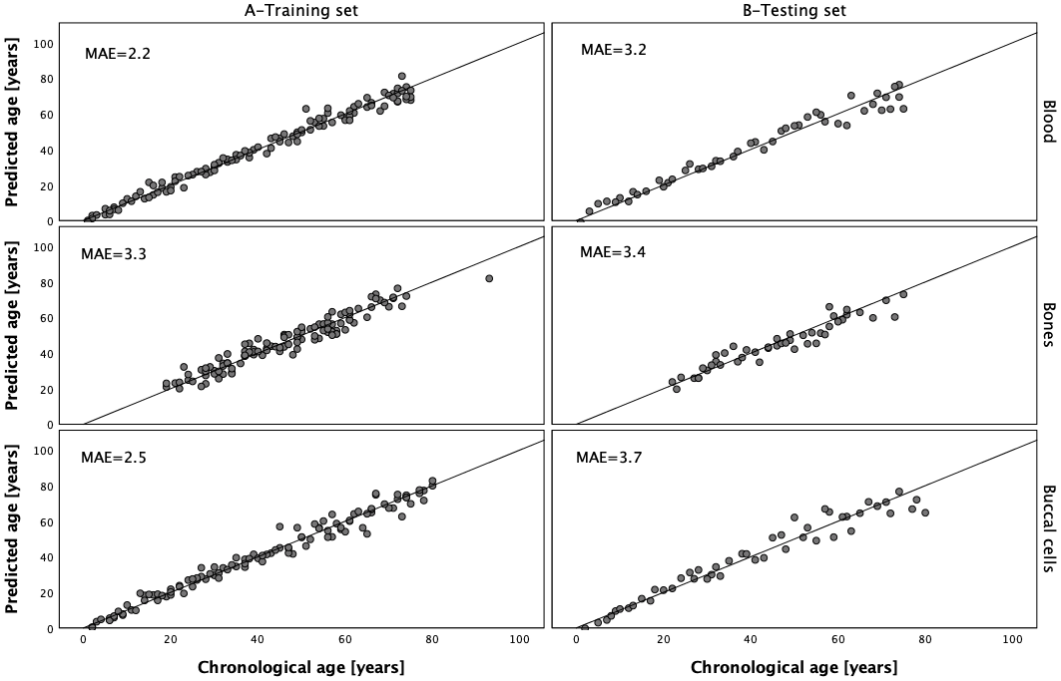
**Predicted vs. chronological age in the blood, bones and buccal cells training and testing sets.**

DNA methylation data generated with the MPS tool for 112 blood samples, 112 buccal cell samples and 112 bone samples were next used to train the three respective models. Because of the lack of the data in *PDE4C* C5 or *ELOVL2* C2, two samples from the training sets were rejected and therefore the final number of samples used to train the models for buccal cells and bones was 111. The multivariate stepwise linear regression method was applied to select markers from the available set of 44 CpGs from eight genes and to train the final models. The data for the selected CpG positions in *ELOVL2* were power transformed before prediction analysis and this treatment proved to improve age prediction for DNA from blood and buccal cells. The analysis of blood samples showed that the optimal age model for blood centered on 6 CpG sites from six genes, *ELOVL2*, *MIR29B2CHG*, *KLF14*, *TRIM59*, *FHL2* and *PDE4C* (Table 3). Effect sizes expressed by β values were different and except for marker *MIR29B2CHG* C1 (β = -0.234), were positively correlated with age. The largest effect size in the model was attributed to the power transformed *ELOVL2* C7 position (β = 0.328; *P*-value = 3.24×10^-7^) and the smallest one to *TRIM59* C8 (β = 0.096; *P*-value = 4.48×10^-4^). This model explains 98.2% of age variation (R^2^) observed in the training set and predicts age with an accuracy of MAE = 2.2 years in the training set and MAE = 3.2 years in the testing set (Figure 5 and Table 4). Table 5 outlines the optimal model for buccal cells comprising 5 CpG sites from 5 genes, *PDE4C*, *MIR29B2CHG*, *ELOVL2*, *KLF14* and *EDARADD*. The largest β value was observed for marker *PDE4C* C5 (β = 0.351; *P*-value = 1.29×10^-7^) and this position was found to explain most of the age variation explained by the model (R^2^ = 93.1%). Power transformed *ELOVL2* C9 had the second largest effect (β = 0.244; *P*-value = 4.81×10^-5^). Negative and weak correlation was observed for a CpG in *EDARADD* not included in the blood model (β = -0.098). The final model explains R^2^ = 97.5% of variation observed in the training set and predicts age with an accuracy of MAE = 2.5 years in the training set and MAE = 3.7 years in the testing set (Figure 5 and Table 4).

**Table 3:** Characteristics of the markers in age predictive model for blood.

**CpG**	**CpG_ID**	**GRCh38 chromosome position**	**Standardized coefficient β**	**t statistic**	***P*-value**	**Adjusted *R*^2^**
*ELOVL2* C7^a^	-	chr6:11044634	0.328	5.458	3.24×10^-7^	0.952
*PDE4C* C5	-	chr19: 18233127	0.125	2.785	0.006	0.962
*MIR29B2CHG* C1	-	chr1:207823681	-0.234	-8.555	1.05×10^-13^	0.974
*KLF14* C4	-	chr7:130734375	0.111	4.751	6.46×10^-6^	0.977
*TRIM59* C8	-	chr3:160450202	0.096	3.624	4.48×10^-4^	0.980
*FHL2* C1	cg06639320	chr2:105399282	0.169	3.419	0.001	0.982

^a^power transformation for the DNA methylation data for *ELOVL2* was applied (y = 0.002*x^2.366) before multiple linear regression analysis.

**Table 4:** MAE in different age categories.

**Age category**	**MAE in blood**			**MAE in buccal cells**			**MAE in bones**	
	**Training set**	**Testing set**		**Training set**	**Testing set**		**Training set**	**Testing set**
1	1.8 (N=28)	2.2 (N=12)		1.7 (N=28)	1.5 (N=12)		3.0 (N=2)^b^	-^b^
2	1.5 (N=28)	1.6 (N=12)		2.0 (N=27)	2.8 (N=12)		3.2 (N=42)	2.8 (N=17)
3	2.7(N=28)	3.5 (N=12)		3.4 (N=28)	5.7 (N=12)		3.3 (N=47)	3.7 (N=21)
4	3.0 (N=28)	5.5 (N=12)		3.1 (N=28)	4.8 (N=12)		3.3 (N=20)	3.9 (N=8)
Overall	2.2 (N=112)	3.2 (N=48)		2.5 (N=111)	3.7 (N=48)^a^		3.3 (N=111)^c^	3.4 (N=46)^c^

Age categories: age category 1: 1–20; age category 2: 21-40; age category 3: 41–60; age category 4: >60.

^a^one sample had missing data in *PDE4C* C5.

^b^there were only two bone samples falling into the age category of 1-20 (both included in the training set) therefore calculation of MAE for the testing set was impossible and the value of MAE designated for the training set should be treated with caution.

^c^one sample from the training set and three from the testing set had missing data in *ELOVL2* C2.

**Table 5:** Characteristics of the markers in age predictive model for buccal cells.

**CpG**	**CpG_ID**	**GRCh38 chromosome position**	**Standardized coefficient β**	**t statistic**	***P*-value**	**Adjusted *R*^2^**
*PDE4C* C5	-	chr19: 18233127	0.351	5.671	1.29×10^-7^	0.931
*MIR29B2CHG* C3	-	chr1:207823672	-0.232	-9.472	1.02×10^-15^	0.955
*ELOVL2* C9^a^	-	chr6:11044628	0.244	4.243	4.81×10^-5^	0.966
*KLF14* C1	cg14361627	chr7:130734355	0.17	5.441	3.54×10^-7^	0.972
*EDARADD* C1	cg09809672	chr1:236394383	-0.098	-3.303	0.001	0.975

^a^power transformation for the DNA methylation data for *ELOVL2* was applied (y = 0.055*x^1.673) before multiple linear regression analysis.

Table 6 shows the optimal model for bones comprising six CpGs from four genes, *ELOVL2*, *PDE4C, KLF14* and *ASPA*. The largest β value was observed for marker KLF14 C3 (β = 0.498; *P*-value = 2.002×10^-16^) and the smallest positive effect size in the model was attributed to marker *ELOVL2* C7 (β = 0.20). The developed prediction model explains R^2^ = 92.4% of variation in age observed in the training set and predicts age with an accuracy of MAE = 3.3 in the training set and MAE = 3.4 in the testing set (Figure 5 and Table 4).

**Table 6:** Characteristics of the markers in age predictive model for bones.

**CpG**	**CpG_ID**	**GRCh38 chromosome position**	**Standardized coefficient β**	**t statistic**	***P*-value**	**Adjusted *R*^2^**
*ELOVL2* C2	cg24724428	chr6:11044655	-0.246	-2.758	0.007	0.735
*ELOVL2* C7	-	chr6:11044634	0.200	2.606	0.010	0.924
*KLF14* C3	-	chr7:130734372	0.498	9.788	2.002×10^-16^	0.850
*PDE4C* C6	cg01481989	chr19:18233131	0.374	4.096	8.349×10^-5^	0.912
*PDE4C* C4	-	chr19:18233105	0.245	2.938	0.004	0.916
*ASPA* C1	cg02228185	chr17:3476273	-0.142	-3.467	0.001	0.920

Our data show an increase in the MAE value with increased age of the sample donors for blood and buccal cell models. The highest MAE value was observed in the 3^rd^ (age 41-60 years) and 4^th^ (>60 years) age categories (Table 4). When age was analyzed as a continuous variable it was significantly correlated with MAE in both tissue types (Pearson correlation *P*-value of 0.001 and 3.86×10^-4^ for blood training and testing sets, respectively and *P*-values of 0.011 and 2.52×10^-4^ for buccal cells training and testing sets, respectively). This effect was not seen in bones, neither in the training (*P*-value = 0.122) nor the testing set (*P*-value = 0.070).

### Age prediction in blood from methylation data obtained with the VISAGE basic tool

To enable age prediction from methylation data collected with the previously reported VISAGE basic tool for age estimation from blood, comprising a 5-plex MPS assay [19], we re-trained our 112-sample containing blood training set for the five CpGs based on data generated with the VISAGE enhanced tool. This model predicts age in the training set with an accuracy of MAE = 2.7 and in the testing set with MAE = 3.8. The lower accuracy achieved with the 5-markler model compared to the full 6-marker model (see above) can be explained by the addition of *PDE4C* in the VISAGE enhanced model. Moreover, when performing age prediction modelling using data for these five markers obtained from buccal cells and bones, we achieved higher errors for buccal cells with MAE = 3.9 and 4.3 for training and testing sets, respectively, and bones with MAE = 4.7 and 4.0 years for training and testing sets, respectively. Notably, the buccal and bone models based on the eight DNA methylation markers of the VISAGE enhanced tool achieved more accurate age prediction than those based on the five markers in the VISAGE basic tool for blood, lacking the three additional markers, covering all three somatic tissues in the VISAGE enhanced tool.

To check the compatibility of the two VISAGE age tools, the VISAGE basic tool for estimating age from blood and the VISAGE enhanced tool for estimating age from somatic tissues including blood, buccal cells and bones, methylation results obtained for the seven artificially methylated DNA standards generated with the 8-plex assay of the VISAGE enhanced tool were compared to those produced with the 5-plex assay of the VISAGE basic tool [19]. Both assays showed very similar amplification bias for DNA methylation quantifications at the overlapping five CpGs (Supplementary Figure 4A). When subtracting methylation levels obtained with the VISAGE 8-plex assay from values obtained for the 5-plex assay, the 5-plex assay of the VISAGE basic tool appeared to produce slightly higher methylation values at the five relevant CpG sites (mean differences = 2.4%) than the 8-plex assay of the VISAGE enhanced tool did. The absolute differences between methylation quantifications at the same expected DNA methylation level were on average 3.0% ± 2.7% across the seven DNA standards and markers for the two assays (Supplementary Figure 4B). To explore whether these differences were significantly different, observed methylation values with the 8-plex enhanced tool assay were plotted against those of the 5-plex basic tool (Supplementary Figure 5). Regression models showed no statistically significant performance differences between the two assays (Bonferroni corrected *P*-values: *ELOVL2* C7: 0.681, *KLF14* C1: 1.000, *MIR29B2CHG* C1: 1.000, *FHL2* C2: 0.756, *TRIM59* C7: 1.000). These results indicate that the blood model developed here for the five CpGs in the VISAGE basic tool for age estimation from blood [19] can be used for estimating age in blood, based on data generated with the VISAGE basic tool. However, due to the lower errors achieved with the blood model based on the 8-plex data, use of the VISAGE enhanced tool, including 8-plex data and the 6-marker predictive model, is advised to estimate age from blood.

### Accuracy of developed predictive models in predicting age in various human tissues

To assess the performance of the developed prediction models in predicting age in human tissues other than blood, buccal cells or bones, we also applied the VISAGE enhanced age tool to 24 DNA samples collected postmortem from various tissues of 22 males and 2 females ranging in age from 21 to 73 years at the time of death (mean age = 41.0 ± 12.5). Table 7 summarizes the results of these experiments. This limited dataset confirmed that the developed blood model can accurately predict age in blood samples of deceased individuals (MAE = 3.1). However, the prediction accuracy obtained with postmortem cartilage and muscle samples estimated with the models individually developed for blood, buccal cells and bones was unsatisfactory (Table 7). Univariate association testing conducted using linear regression in this small set of 24 samples showed positive signals of association for *TRIM59*, *FHL2*, *MIR29B2CHG*, *ELOVL2* and *PDE4C* in muscle and cartilage (data not shown). Although we found our eight DNA methylation markers were associated with age in the vast majority of CpG sites in different tissue types, detailed analysis of the DNA methylation-age correlation for particular CpG sites showed a different pattern of DNA methylation changes in different tissues (Supplementary Figure 6), and this underlies the high predictive error observed for muscle and cartilage when using the developed models.

**Table 7:** Age prediction accuracy in blood, cartilage and muscle samples obtained with the developed models for blood, buccal cells and bones.

**DNA source**	**N**	**MAE**		
		**Blood age model**	**Buccal cells age model**	**Bone age model**
Blood	24	3.1	6.5	4.9
Cartilage	24	13.1	12.3	25.8
Muscle	24	17.1	17.3	13.7

## DISCUSSION

Although the use of hundreds of CpGs for age estimation delivers small prediction errors [2, 7], such marker densities are currently impossible in forensic applications due to the lack of suitably sensitive DNA technology. Smaller sets of DNA methylation age markers that can be analyzed with forensically suitable technology typically predict age in different tissues with different accuracies. To develop a universal epigenetic age prediction tool for forensic applications, the VISAGE enhanced tool for age estimation from somatic tissues represents a significant step forward in age estimation for criminal investigations. This tool includes a MPS-based assay for eight DNA methylation markers (44 CpGs) and three different statistical models appropriate for blood, buccal cells and bones as DNA sources. The eight markers previously demonstrated age correlation in various forensically relevant DNA sources [12, 13, 15, 28, 30, 33]. The data we used here to train and test the prediction models were generated with the developed MPS tool, as method-to-method bias prevents the usage of datasets available from the literature.

A crucial step in the optimization of the MPS assay of the VISAGE enhanced age tool was the development of the multiplex PCR for bisulfite converted DNA. The severe chemical treatment during bisulfite conversion not only leads to DNA degradation and loss, but also markedly reduces the complexity of the DNA sequence. Consequently, primer specificity is potentially reduced and the formation of primer dimers is favored [34]. Additionally, most targeted CpGs were located in CpG islands (except *ASPA* and *EDARADD*) that represent regions difficult to amplify. We successfully combined *ASPA*, *EDARADD* and *PDE4C* with the five markers of the VISAGE basic assay (*ELOVL2*, *MIR29B2CHG*, *KLF14*, *FHL2, TRIM59)* into an 8-plex PCR assay for the final VISAGE forensic MPS tool described here. Samples processed with the re-configured assay using the MiSeq Reagent Kit v3, which offers increased read length and is commercially available in forensic quality, yielded high quality sequences and high read depths for all eight amplicons. The observed variability between read depths at targeted CpGs most likely results from differences in primer pair efficiencies that could not be fully balanced by adjusting primer concentrations (Figure 1A, 1B). Assay performance was confirmed with DNA methylation standards that allowed the assessment of methylation quantification for robustness and linearity. Since the introduction of bisulfite conversion for DNA methylation analysis, PCR bias towards the unmethylated or methylated template molecule has been repeatedly described [35–37], highlighting the difficulties in achieving a completely methylation-independent amplification. To avoid accentuating such bias, mismatch primers were used for CpG sites within primer sequences. Although methylation quantification of most markers was close to the line of identity, we observed a bias towards methylated DNA templates (*MIR29B2CHG* and *EDARADD)* as well as an underestimation of methylation levels (*ELOVL2*). Interestingly, the avoidance of a CpG or the inclusion of one or two CpGs in the primer sequences did not appear to noticeably change the strength of methylation bias. The observed PCR bias appeared stable, with minor differences between duplicates (mean = 1.9% ± 1.2%), which is important for reliability of age prediction. The most challenging marker for multiplex development was *PDE4C*, for which a higher variability (4.8% ± 3.7%) was observed in comparison to other markers. Nevertheless, methylation quantification of samples used for predictive modelling showed that *PDE4C* has a wide DNA methylation range and a large effect size, which makes it a reliable marker for all three age prediction models. Assuming that the range of DNA methylation throughout a person’s lifespan determines the required minimum accuracy for a marker, the highest accuracy would be needed for *KLF14*. In line with this consideration, the methylation quantification at optimum DNA input for *KLF14* showed the smallest differences between duplicates. For further analysis of the used DNA methylation controls, we compared maximum differences in methylation values obtained at the same target and sample. Surprisingly, the highest variability was observed at 100% methylation level (3.8% ± 2.6%) and not, as expected at 50% methylation. As the fully methylated DNA standards represent an artificial system, some variability may be inherent in the methylation controls.

Like all quantitative methods, DNA methylation analysis to attempt chronological age prediction is impacted by stochastic effects when the DNA input amount is low [38]. This poses a limitation to the application of quantitative DNA methylation to forensic samples that contain only minute amounts of DNA. Furthermore, the DNA is not necessarily equally distributed in such tissues, which adds additional variation due to a sampling effect. Additionally, DNA loss during bisulfite conversion and further stochastic processes during the subsequent multiplex PCR step count up to this variation. Previous studies suggested that 20 ng to 10 ng DNA template used for PCR are required for a reliable methylation quantification [38, 39], although a higher sensitivity (10 ng DNA input for bisulfite conversion ~2 ng at PCR) has recently been reported [26]. We tested a dilution series of the initial DNA amount used for bisulfite conversion to perform a first sensitivity evaluation of the VISAGE enhanced tool age assay. When comparing mean quantified DNA methylation levels to the optimum DNA input, we observed a robust quantification down to 20 ng DNA input for most markers. According to previous studies investigating DNA loss during bisulfite conversion, the DNA amount used for PCR would be estimated to be from 8.8 ng (45% DNA loss [40]) to 11.8 ng (26% DNA loss [41]), which is in agreement with considerations regarding low DNA quantities. Results of this sensitivity study clearly indicate that methylation quantification of 1 ng samples was unreliable with increased differences to the reference DNA input as well as an increased variability between targeted CpGs of the same amplicon. Additionally, samples at 1 ng DNA input showed CpGs that appeared completely methylated (e.g. at *ELOVL2*, *PDE4C*) or unmethylated (e.g. *PDE4C*), indicating an amplification bias towards the methylated or unmethylated DNA template during PCR. Overall, the results from development and optimization of the VISAGE enhanced tool for age estimation from somatic cells showed promising results for future application in routine forensic DNA analyses. However, small differences between sequencing runs, may affect the final age prediction outcome, as recently described by Han et al. (2020) [42]. The study reported a shift in DNA methylation levels between sequencing runs that led to an increased MAE in an independent validation set. The extent of such variabilities and the impact on age prediction, particularly when analyzing low DNA input samples, needs to be further addressed before application in real casework. Further validation studies including inter-laboratory exercises will bring deeper insight into the assay’s robustness, reproducibility and sensitivity.

The developed 8-plex MPS assay was used to collect DNA methylation data necessary for the development of the three age prediction models. The model for blood comprises six CpGs from *ELOVL2*, *MIR29B2CHG*, *KLF14*, *FHL2*, *TRIM59* and *PDE4C* and predicts age with MAE of 3.2 years. As expected, *ELOVL2* was the top ranked marker, which alone explains 95% of variation in this age model. Non-linear age-DNA methylation correlation was observed in this DMRs, in line with other studies [28, 43]. The correlation of DNA methylation in *ELOVL2* with age in blood was first suggested by Garagnani et al. (2012) [44] and was soon confirmed in independent studies, making *ELOVL2* the most important epigenetic age predictor in a range of fields including forensics [3, 4, 39, 45]. The five remaining predictors include blood markers widely validated in studies of different populations from Europe and Asia [13–15, 22, 30, 46].

The model for buccal cells includes five CpG sites from *PDE4C*, *ELOVL2*, *MIR29B2CHG*, *KLF14* and *EDARADD* and predicts age with a MAE of 3.7 years. *PDE4C* was the top ranked marker and alone explained 93.1% of variation in age. Early studies suggested *PDE4C* as an age predictor in blood and saliva [27, 47] and it was rapidly adopted in age prediction models for blood [11, 28]. In our study, this marker had a higher predictive value in buccal cells, which confirms the conclusions of the study of Eipel et al. (2016) [31], whose markers showed higher correlation with age in saliva and buccal cells than in blood. *ELOVL2*, *MIR29B2CHG* and *KLF14* come from the five best predictors for blood selected by the study of Zbieć-Piekarska [12], which had been shown to be suitable for predicting age in saliva and buccal cells in Asian populations [15]. The buccal cell tissue marker *EDARADD*, was included in the first age prediction algorithm developed for saliva [27] and replicated in other studies that investigated blood, saliva and buccal cells [28, 48–51].

Furthermore, we present here an age prediction model for bones which is based on only six CpGs from four DMRs in *ELOVL2, KLF14, PDE4C* and *ASPA*. This model predicts age with a relatively small error, with a MAE of 3.4 years in the testing set. Age prediction attempts using epigenetic markers in bone material are rare. Prediction models reported for teeth were based on a relatively small number of samples and found *ELOVL2, PDE4C, EDARADD, FHL2* and *PENK* to be useful predictors of age in teeth [16, 18, 28]. Naue et al. (2018) attempted to predict age in various tissues including bone and found DNA methylation at *ELOVL2, KLF14* and *TRIM59* to be correlated with age in bones. Other suggested age predictors for bones include *DDO, F5, LDB2, NKIRAS2, RPA2* and *ZYG11A* [13]. In a recent paper Lee et al. (2020) reported *TMEM51* and *EPHA6* as new age markers for bones identified from Infinium MethylationEPIC BeadChip array data. This study also confirmed age correlation in bones for *ELOVL2, FHL2, KLF14* and *TRIM59* [17].

Our study shows that the eight CpGs selected for predicting age in somatic cells are a robust set of markers for developing accurate age prediction algorithms for DNA extracted from blood, buccal cells and bones. In particular, *PDE4C*, *ELOVL2* and *KLF14* are used in all models and various combinations of just eight markers can predict age in the three tissues with effective accuracy with a MAE of 3.2 - 3.7 years.

In agreement with other studies, an increased MAE in age predictions of older individuals was observed in blood and buccal cells, which can be explained by a combination of genetic and environmental factors influencing the individual rate of aging [6, 43, 52]. Therefore, we calculated the MAE for different age groups, which allows to account for the corresponding age category in the interpretation of real casework. In addition, the MAE range can be provided along with the predicted age (e.g. from 2.2 to 5.5 years for blood).

Our study confirms the importance of *ELOVL2* and *PDE4C* for epigenetic age prediction and provides further evidence that *MIR29B2CHG* (ranked 3^rd^ in the blood model and 2^nd^ in the buccal cell model), is a valuable age predictor in forensics. In the first two markers, hypermethylation with age is observed, and in the third hypomethylation. All three are characterized by a wide range of DNA methylation levels during an individual’s lifespan (50-70%). *KLF14* is characterized by the lowest range of DNA methylation over a lifespan but this marker is consistently suitable in all three predictive models [21, 51, 53]. *ASPA* was chosen only for use as a bone age predictor in our study, while *FHL2* and *TRIM59* were selected exclusively as blood age predictors, although correlation with other tissue types has been demonstrated for these markers. Our study aimed to select a universal set of DMSs for epigenetic age prediction in various somatic tissues, but since the pattern of DNA methylation change differed in various cell types tested, the DNA methylation data collected for individual tissues had to be incorporated into separate training sets for tailored prediction models. In addition, our pilot study of postmortem samples, which analyzed tissues from 24 deceased persons, indicated the models developed did not predict cartilage and muscle age correctly.

In summary, the study outlined here presents a complete tool for estimating a person’s age from DNA in forensic applications that deal with low amounts of DNA from the three forensically relevant tissue types of blood, buccal cells and bones. The VISAGE enhanced tool for age estimation in somatic tissues comprises a single bisulfite MPS assay targeting 44 CpGs from eight carefully selected DNA methylation markers and three separate predictive models for blood, buccal cells and bones. The MPS assay provided reliable and reproducible methylation quantifications, enabling accurate age prediction in samples down to a minimum of 20 ng of DNA. The three individual tissue models provide a good balance of marker number and accuracy given the capacity limitations of the DNA methylation measurement technology used. Future work could focus on increasing the model testing datasets to investigate the reliability of reported error estimates for the three models. It will also be useful to gauge the performance of the age prediction models for data produced using the VISAGE enhanced tool with additional forensically relevant somatic tissues. Notably, DNA methylation variation in non-somatic tissues, such as semen, is known to differ from that in somatic tissues; the development of an epigenetic tool for age estimation in semen is currently in progress by the VISAGE Consortium.

## MATERIALS AND METHODS

### Selection of DNA methylation markers for age prediction in somatic tissues

Five age markers previously described in Zbieć-Piekarska et al. (2015) [12] were used as the basis for developing the VISAGE enhanced tool for age estimation of forensic DNA from somatic cells. We performed a comprehensive literature search and extended the original marker set comprising *ELOVL2*, *MIR29B2CHG*, *TRIM59*, *KLF14* and *FHL2* with the three additional markers of *EDARADD*, *PDE4C* and *ASPA*. It has been shown in multiple studies that these three markers have considerable capacity to further improve prediction of age in buccal cells/saliva and have been demonstrated to correlate with age in other somatic cells including bones [11, 12, 27–31]. The eight marker combination selected for inclusion in the expanded VISAGE MPS multiplex comprised a total of 44 individual CpG sites (Supplementary Table 1).

### Assay design and development

### Multiplex PCR

Development of the multiplex PCR assay for targeted bisulfite sequencing used primer designs established for the VISAGE basic test [19] (*ELOVL2*, *KLF14*, *TRIM59*, *FHL2* and *MIR29B2CHG)* with primers for the three additional markers (*PDE4C*, *ASPA* and *EDARADD*), either newly designed using MethPrimer [54] and PrimerSuite [55] or gathered from the literature. When CpG sites within the primer sequences were unavoidable, a deliberate mismatch was introduced. In the specific cases of *PDE4C* and *MIR29B2CHG*, degenerate primers carrying a Y at CpG positions were also designed and tested, but no increase in amplicon yield was observed (data not shown) and consequently, mismatch primers were utilized in the final multiplex PCR. All newly tested primer pairs are listed in Supplementary Table 3. The formation of non-specific PCR products and primer dimers was evaluated *in silico* using BiSearch [34] and AutoDimer [56]. Primer sequences and final multiplex PCR concentrations are listed in Table 1.

PCR optimizations were performed with DNA extracted from 10 ml EDTA venous whole blood from three samples using the Blood Maxi Kit (Qiagen, Hilden, Germany) and quantified by real-time quantitative PCR [57]. Blood samples either derived from one individual sampled within this study under written informed consent (approved by the ethics commission of the Medical University of Innsbruck under study number 1086/2017) or were purchased from Biotrend (Köln, Germany). Bisulfite conversion was performed with 200 ng extracted DNA using the Premium Bisulfite Kit (Diagenode, Ougrée, Belgium) according to the manufacturer’s protocol. An additional dry spin before elution was performed to prevent ethanol carry-over into the PCR. A total of 2 μl converted DNA was used for primer tests with the Multiplex PCR Kit (Qiagen) in 25 μl assay volume. Annealing temperature gradient PCRs were performed to optimise the singleplex reactions to test primers targeting *ASPA*, *PDE4C* and *EDARADD* as well as with the entire multiplex system. Post-PCR purification was performed with 1.5X volume of AMPure XP beads (Beckman Coulter, Brea, California, USA) and 15 μl low TE (10 mM Tris, 0.1 mM EDTA, pH 8) were used for elution. PCR products were evaluated for amplicon yield and size on the Bioanalyzer using the DNA 1000 Kit (both Agilent Technologies, Santa Clara, CA, USA). The final multiplex PCRs were carried out using the thermocycler steps: initial denaturation at 95° C for 15 min; 38 cycles of 95° C for 10 s, 57° C for 30 s, 72° C for 30 s; final elongation at 72° C for 10 min.

PCR products were also assessed with Sanger sequencing to verify all amplicons before massively parallel sequencing as described in [58]. In brief, reactions were carried out using BigDye Terminator v1.1 Cycle Sequencing kit (Thermo Fisher Scientific - TFS, Waltham, MA, USA) in 10 μl reaction volumes and 0.3 μM primer (listed in Table 1). The thermal cycling comprised steps: 96° C for 1 min; 25 cycles of 95° C for 15 s, 50° C for 5 s and 60° C for 4 min. Cycle sequencing products were purified using centrifugation over Sephadex G-100 columns (Amersham, Little Chalfont, UK). Electrophoresis of sequencing products was performed on an ABI3500 instrument (TFS) using standard settings. Raw sequences were analysed with the Sequencer 5.1 (Gene Codes Corporation, Ann Arbor, MI, USA) software and assembled with an in-house prepared reference (bisuflite converted reference for targeted amplicons).

### Massively parallel sequencing and data analysis

All PCR products were quantified using the Qubit dsDNA HS Assay Kit (TFS) for library preparation. All protocol steps were performed in half volume with 50 ng purified PCR products using the KAPA Hyper Prep Kit with KAPA Library Amplification Primer Mix and KAPA SI Adapter Kit Set A+B at 15 μM (all Roche, Basel, Switzerland), following the manufacturer’s instructions. Post-ligation and post-amplification clean-ups were performed with 0.8X and 1X AMPure XP beads, respectively. Libraries were amplified with 5 PCR cycles. Purified libraries were quantified with the KAPA Library Quantification Complete kit (Roche) and evaluated using the DNA 1000 Kit on the Bioanalyzer. For sequencing, libraries (N = 24 per run) were pooled equimolarly (4 nM) and processed according to the MiSeq System Denature and Dilute Libraries Guide, Protocol A (Document #15039740 v10; Illumina, San Diego, CA, USA). All libraries were diluted to 7 pM and spiked with 2 μl 20 pM PhiX control v3. For assay optimization, sequencing was performed with the MiSeq Reagent Kit v2 2x 150 cycles or the MiSeq Reagent Kit v3 2x 200 cycles (both Verogen, San Diego, CA, USA).

### Assay re-optimization (final design)

In order to balance amplicon yields, *PDE4C* primers were tested at increasing concentrations (0.4 μM, 0.6 μM, 0.8 μM, 1 μM). Multiplex PCR was performed with 8 μl eluate from bisulfite conversion of 200 ng DNA, followed by library preparation according to the protocol described above. Final libraries were evaluated on the Bioanalyzer using the DNA 1000 Kit (Supplementary Figure 2). The newly optimized multiplex PCR was tested using the MiSeq Reagent v3 kit, which allows for longer read lengths (v3: 600 cycles versus v2: 300 cycles) and provides higher output (v3: 13.2 to 15 GB versus v2: 4.5 to 5.1 GB).

### Performance evaluation with DNA standards of known methylation state

Assay evaluation was performed with artificially methylated DNA standards, which were prepared using the human WGA methylated and non-methylated DNA Set (Zymo Research, Irvine, CA, USA). Fully methylated and non-methylated control DNA samples were diluted to 20 ng/μl in low TE and quantified with the Qubit dsDNA HS Assay Kit (TFS). These two control DNA dilutions were mixed at different volume proportions to achieve 5%, 10%, 25%, 50% and 75% methylated DNA standards. DNA inputs stated for assay evaluation refer to the DNA amount used for bisulfite conversion. The optimum DNA input (200 ng) is indicated by the manufacturer of the Premium bisulfite kit. The whole eluate from bisulfite conversion could be used for the multiplex PCR to increase sensitivity however, to ensure equal volumes within the performance evaluation, 8 μl of eluate were used for amplification. DNA methylation standards at optimum input were used to test the first assay design (duplicates of 0%, 25%, 50%, 75% and 100% methylated DNA standards) as well as the re-optimized protocol (design 2; duplicates of 5%, 10%, 25%, 50%, 75% methylated DNA standards and one replicate of 0% and 100% methylated control). Sensitivity assessment of the re-optimized protocol was performed with 200 ng, 100 ng, 50 ng, 20 ng, 10 ng and 1 ng DNA input of a 50% methylated DNA standard. Samples were processed together with negative template controls (NTC, PCR grade water) for all steps with two NTCs selected for sequencing. Sequencing baseline noise was below the 1,000 reads threshold at all amplicons for NTC-1 (mean = 169.9 ± 89.1 paired reads). NTC-2 (mean = 747.5 ± 972 paired reads) showed higher read depth at *KLF14* C1 and C2 (mean = 5702.5 paired reads) and *TRIM59* C1 to C3 and C8 (1,064 paired reads). Inspection in IGV showed misaligned reads at *KLF14* causing high read depth (Supplementary Figure 3A), whereas a low level of contamination at *TRIM59* cannot be fully excluded. However, read depth was very low compared to the overall read depth at this amplicon (overall mean = 141,623.1 ± 33,757.9 paired reads).

### Development of predictive models

### Samples used in predictive modelling

Peripheral blood was collected in EDTA-tubes from 160 unrelated, healthy individuals: 80 males and 80 females in the age range 1–75 years (mean 40.2 ± 22.7) under two research projects AEVITAS (DOBR/0002/R/ID1/2012/) and NEXT (DOB-BIO7/17/01/2015). These sampling regimes were approved by the Commission of Bioethics at the Institute of Cardiology in Warsaw (IK-NP-0021-79/1396/13) and the Ethics Committee of the Jagiellonian University in Krakow (KBET/122/6120/11/2016); plus 1072.6120.24.2017 for retrospective analysis of samples. In all cases informed consent was provided by participants or their legal representatives (parents). Buccal swabs from 160 unrelated, healthy individuals: 80 males and 80 females in the age range 2–80 years (mean 40.6 ± 22.8) were obtained from a previous EUROFORGEN project (7PR UE, grant no 28548) with the consent of the Commission on Bioethics of the Regional Board of Medical Doctors in Krakow for retrospective analysis of samples (48 KBL/OIL/2008; OIL/KBL/23/2017). Samples were divided into training and testing sets for statistical analyses as presented in Table 2. A set of 161 bone samples (occipital bone or femoral shaft): 129 males and 32 females in the age range 19–93 years (mean 46.1 ± 14.8) was collected during routine autopsies, performed by a forensic medical examiner at the Department of Forensic Medicine, Medical College of Jagiellonian University in Krakow. In addition, blood, muscle (rectus abdominis muscle) and costal cartilage were collected from 24 individuals. The samples were stored at -80° C until further processing. The time from death to autopsy ranged from 1 to 5 days. The study was approved by the ethics committee of the Jagiellonian University in Krakow, Poland (KBET/122.6120.86.2017).

### DNA extraction and quantification

DNA from blood was extracted using a modified salting out procedure [59], PrepFiler Express™ Forensic DNA Extraction Kit (TFS) or standard phenol-chloroform method. Previously used DNA extracts were stored frozen, at 4° C or room temperature (the percentage of methylation detected from different storage conditions was checked randomly and compared with previous Pyrosequencing results) [12]. DNA from buccal swabs and postmortem samples including bones was extracted using a silica-based method with Sherlock AX kit (A&A Biotechnology, Gdansk, Poland). Bone surfaces were cleared of soft tissue with a sterile scalpel and the entire exterior was abraded with a grindstone attached to a Dremel rotary tool to remove potential contaminants. Before the extraction bone pieces (~1cm^3^) were treated with 15% bleach for 1min, repeatedly shaken with 100% ethanol and distilled water (dH2O), and finally subjected to UV irradiation. The thoroughly dried samples were pulverized using a FreezerMill 6750 apparatus (Spex CertiPrep, NJ, USA) and EDTA decalcification applied to each of the samples. DNA concentration was measured in all samples using Qubit dsDNA HS Assay Kit with the Qubit instrument.

### Bisulfite sequencing of samples using the VISAGE assay

DNA from blood was subjected to bisulfite conversion (BC) using the Qiagen 96-well bisulfite conversion kit (Qiagen, Hilden, Germany). In most blood samples, 2,000 ng of DNA was used, and elution was made in 100 μl of elution buffer. In 27 samples with lower DNA concentration (400 ng or less) the elution volume was reduced to 40 μl. Bisulfite conversion of DNA extracted from buccal swab samples and the 233 postmortem samples (161 bone samples and 72 tissues samples) was conducted with the EZ DNA Methylation-Direct Kit (Zymo Research). In all swab samples, DNA input for bisulfite conversion was 200 ng in an elution volume of 10 μl. In samples collected postmortem, the DNA input for BC was 500 ng and the elution volume was 25 μl. DNA methylation data was collected for all samples using the VISAGE assay. Each PCR reaction contained 5 μl bisulfite converted DNA except for samples with lower DNA concentration when 8 or 10 μl BC DNA were used for the PCR amplification. MPS sequencing was performed on the Illumina MiSeq FGx instrument with the MiSeq FGx ForenSeq Reagent Kit, MiSeq Reagent Kit v2 300 cycles and MiSeq Reagent Kits Nano 300 cycles with 5% PhiX Control (except for the first experimental run, which used 1% PhiX Control). The final DNA pool was diluted to 7-12 pM, depending on the run and tissue type. Pools were made from 40 to 74 libraries (including 0% and 100% DNA methylation controls) combined. The MiSeq instrument was set to perform paired-end sequencing of 151 reads in both directions and to complete the data collection, seven main and five additional sequencing runs were performed (Supplementary Table 4).

### Data analysis

### MPS data analysis

Alignment was carried out relative to a custom reference genome containing only the targeted sequences (Supplementary Table 5) using an adapted Burrows-Wheeler alignment for bisulfite converted DNA sequences – bwa-meth [60]. An additional quality control step was performed on the raw data (fastq) for the samples used for predictive modelling, which was reviewed in detail with FastQC software [61]. Bam file creation, sorting, filtering and indexing was performed with Samtools [62]. Alignments of all samples were inspected using the Integrative Genomics Viewer (IGV) [63]. Total numbers of read information was extracted from amplicon positions using bam-readcount with minimum mapping quality and minimum base quality thresholds set to 30 [https://github.com/genome/bam-readcount]. At target CpG sites, obtained C reads were divided by the sum of C reads and T reads to calculate beta values. Observed methylation values refer to percentage beta values. Bisulfite conversion efficiency of samples was estimated by calculating the percentage of mean reversed beta values from all non-CpG-Cs (T reads divided by the sum of C reads and T reads). Total coverage refers to the sum of the number of reads per amplicon (one CpG site per amplicon was selected). Read depth was normalized by dividing the read depth at target positions by the total coverage. Only CpG sites with the minimum number of 1,000 reads were accepted for further analyses including the prediction modelling that was then applied to the data. For statistical analyses Microsoft Excel and R [https://www.r-project.org/] [64] were used.

### Statistical analysis and prediction modelling

The correlation between age and DNA methylation levels at the 44 investigated CpGs in eight genes was analyzed in a training set of 112 carefully selected DNA samples from blood, 112 samples from buccal cells and 112 samples from bones (Table 2). The effect size of particular loci was defined with standardized regression coefficients (β). The linearity of DNA methylation-age correlation was verified for all the tested CpGs. A clear non-linear pattern of correlation was noted for *ELOVL2*, which is in agreement with previous studies [28, 43] and therefore, DNA methylation data for this marker were power transformed before multivariate linear regression analysis was applied. The proportion of age variance explained by individual predictors and their cumulative impact was assessed based on the calculation of R^2^ coefficients. The same datasets of 112 blood, 111 buccal cells samples and 111 bone samples were used to develop linear regression age prediction models. The selection of a set of optimal markers was performed by stepwise linear regression with probability of F statistic, based on a statistical test of the improvement in model error, used as a criterion for predictors entry/removal. The developed models were further tested using an independent set of 48 blood samples, 48 buccal cells samples and 49 bone samples (Table 2). The potential applicability of the developed models to predict age in some other human cell types was verified in a study involving three tissue types collected from 24 deceased individuals (aged 21-73). This experiment involved blood, cartilage and muscle samples. All the analyses were conducted using PS IMAGO PRO 5.1 software (IBM SPSS Statistics 25).

### DNA methylation data comparison to the VISAGE basic assay

Data generated using the VISAGE basic prototype tool from 0%, 5%, 10%, 25%, 50%, 75% and 100% methylated DNA standards (Run1; 200 ng DNA input to bisulfite conversion using the Premium bisulfite kit [19]) was re-analyzed with bam-readcount. The mean methylation values from duplicates were used to calculate the differences between the two assays and to test for statistically significant differences between the two assays. Statistical testing was carried out in R using the “linearHypothesis” function implemented in the package “cars”. To control for the family-wise error in multiple hypothesis testing, *P*-values were adjusted using the Bonferroni method.

## Supplementary Material

Supplementary Materials

Supplementary Figures

Supplementary Table 1

Supplementary Table 2

Supplementary Table 3

Supplementary Table 4

Supplementary Table 5
