# Supplementary material for: Development of the VISAGE enhanced tool and statistical models for epigenetic age estimation in blood, buccal cells and bones
Source: Aging (Albany NY). 2021 Mar 11;13(5):6459–84. doi: 10.18632/aging.202783 (PMC7993733; doi:10.18632/aging.202783)
Supplement: Supplementary Materials [file aging-13-202783-s001.pdf]

SUPPLEMENTARY MATERIALS

Appendix

Centres and investigators of the VISible Attributes through GEnomics (VISAGE) Consortium (http://www.visage-h2020.eu/):

- Erasmus University Medical Center Rotterdam (Netherlands): Manfred Kayser, Vivian Kalamara, Arwin Ralf, Athina Vidaki.
- Jagiellonian University (Poland): Wojciech Branicki, Ewelina Pośpiech, Aleksandra Pisarek.
- Universidade de Santiago de Compostela (Spain): Ángel Carracedo, Maria Victoria Lareu, Christopher Phillips, Ana Freire-Aradas, Ana Mosquera-Miguel, María de la Puente.
- Medizinische Universität Innsbruck (Austria): Walther Parson, Catarina Xavier, Antonia Heidegger, Harald Niederstätter.
- Universität zu Köln (Germany): Michael Nothnagel, Maria-Alexandra Katsara, Tarek Khellaf.
- King's College London (United Kingdom): Barbara Prainsack, Gabrielle Samuel.
- Klinikum der Universität zu Köln (Germany): Peter M. Schneider, Theresa E. Gross, Jan Fleckhaus.
- Bundeskriminalamt (Germany): Ingo Bastisch, Nathalie Schury, Jens Teodoridis, Martina Unterländer.
- Institut National De Police Scientifique (France): François-Xavier Laurent, Caroline Bouakaze, Yann Chantrel, Anna Delest, Clémence Hollard, Ayhan Ulus, Julien Vannier.
- Netherlands Forensic Institute (Netherlands): Titia Sijen, Kris van der Gaag, Marina Ventayol-Garcia.
- National Forensic Centre, Swedish Police Authority (Sweden): Johannes Hedman, Klara Junker, Maja Sidstedt.
- Metropolitan Police Service, London (United Kingdom): Shazia Khan, Carole E. Ames, Andrew Revoir.
- Centralne Laboratorium Kryminalistyczne Policji (Poland): Magdalena Spólnicka, Ewa Kartasińska, Anna Woźniak.
