# Supplementary material for: Development of the VISAGE enhanced tool and statistical models for epigenetic age estimation in blood, buccal cells and bones
Source: Aging (Albany NY). 2021 Mar 11;13(5):6459–84. doi: 10.18632/aging.202783 (PMC7993733; doi:10.18632/aging.202783)
Supplement: Supplementary Figures [file aging-13-202783-s002.pdf]

SUPPLEMENTARY FIGURES

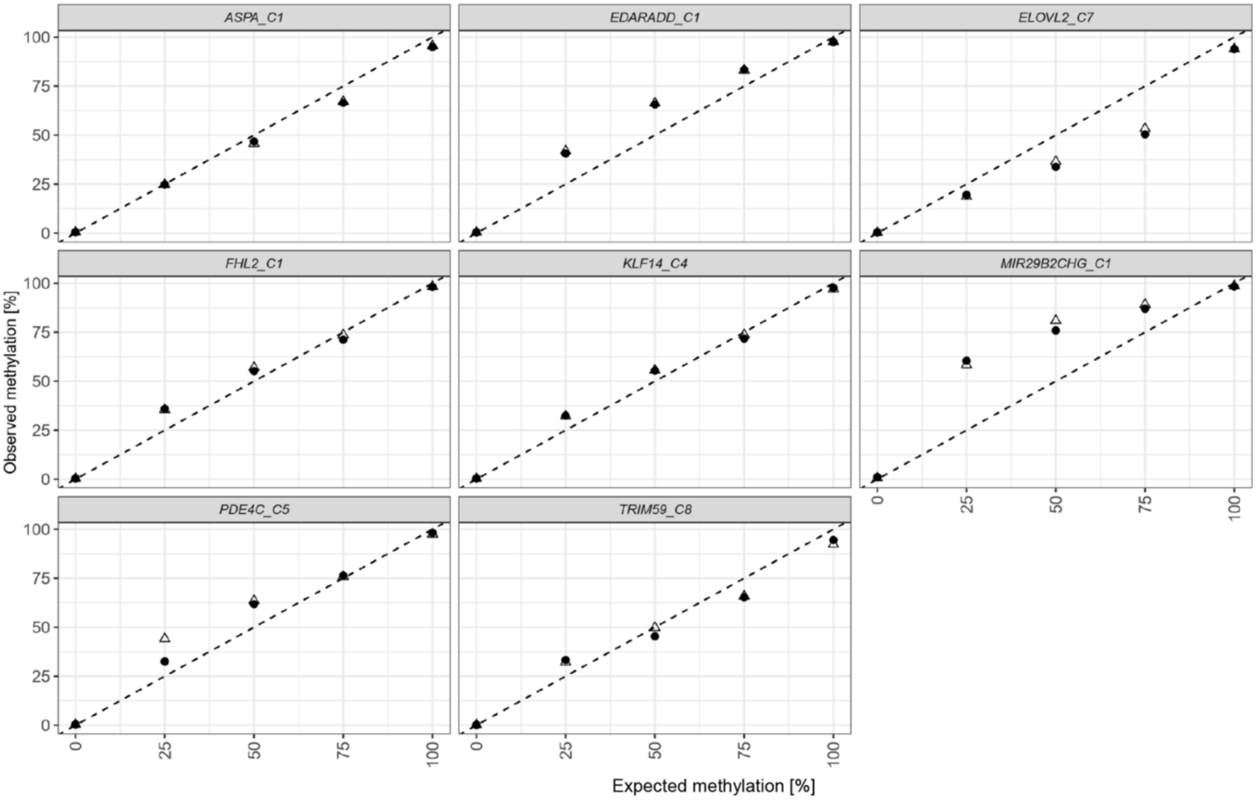

Supplementary Figure 1. Measured versus expected methylation values as obtained for duplicates that were prepared according to the first assay design. The dashed line indicates the line of identity (intercept = 0, slope = 1).

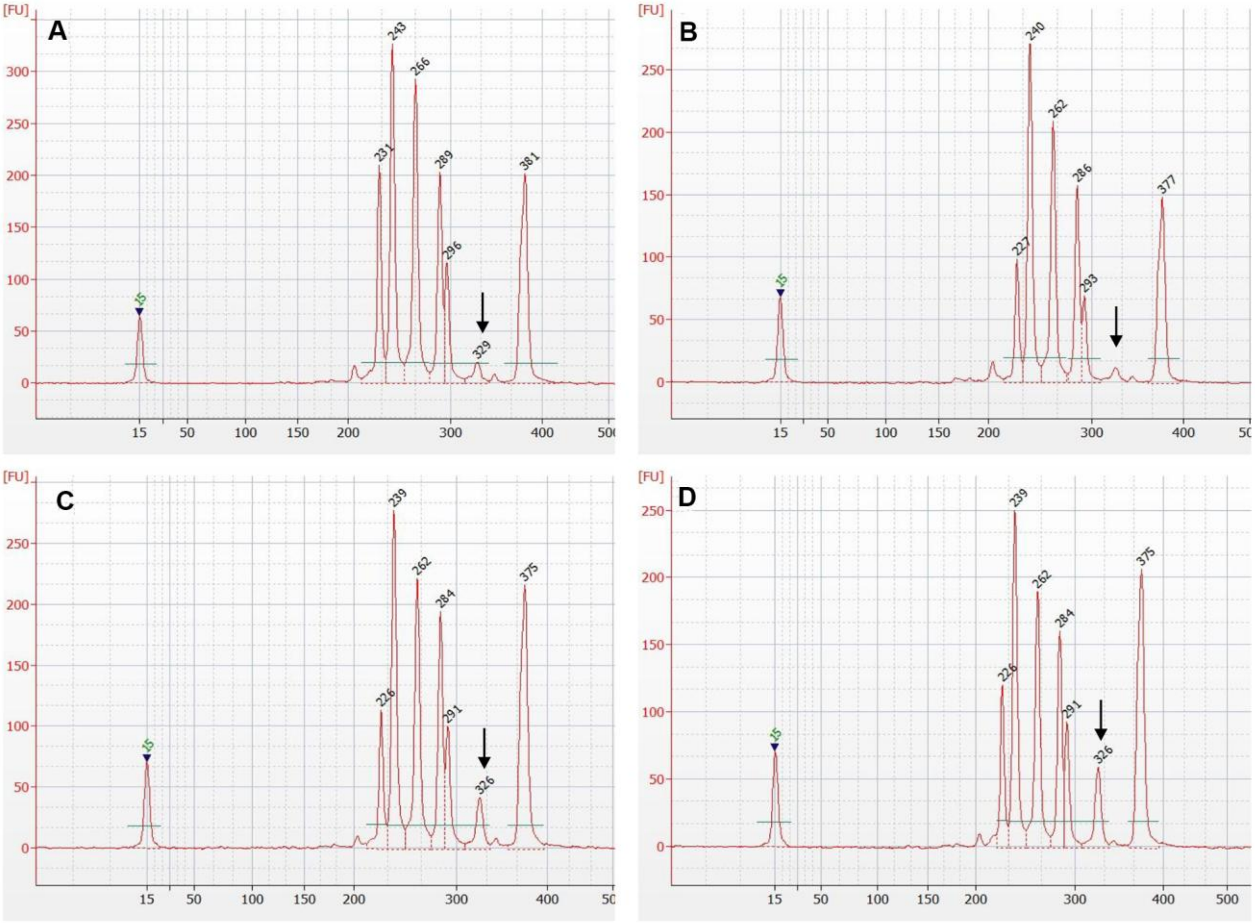

Supplementary Figure 2. Bioanalyzer electropherograms (DNA 1000 kit) show final libraries after multiplex PCR with increasing *PDE4C* concentrations: (**A**) 0.4 µM, (**B**) 0.6 µM, (**C**) 0.8 µM, (**D**) 1 µM. The *PDE4C* amplicon is marked with an arrow.

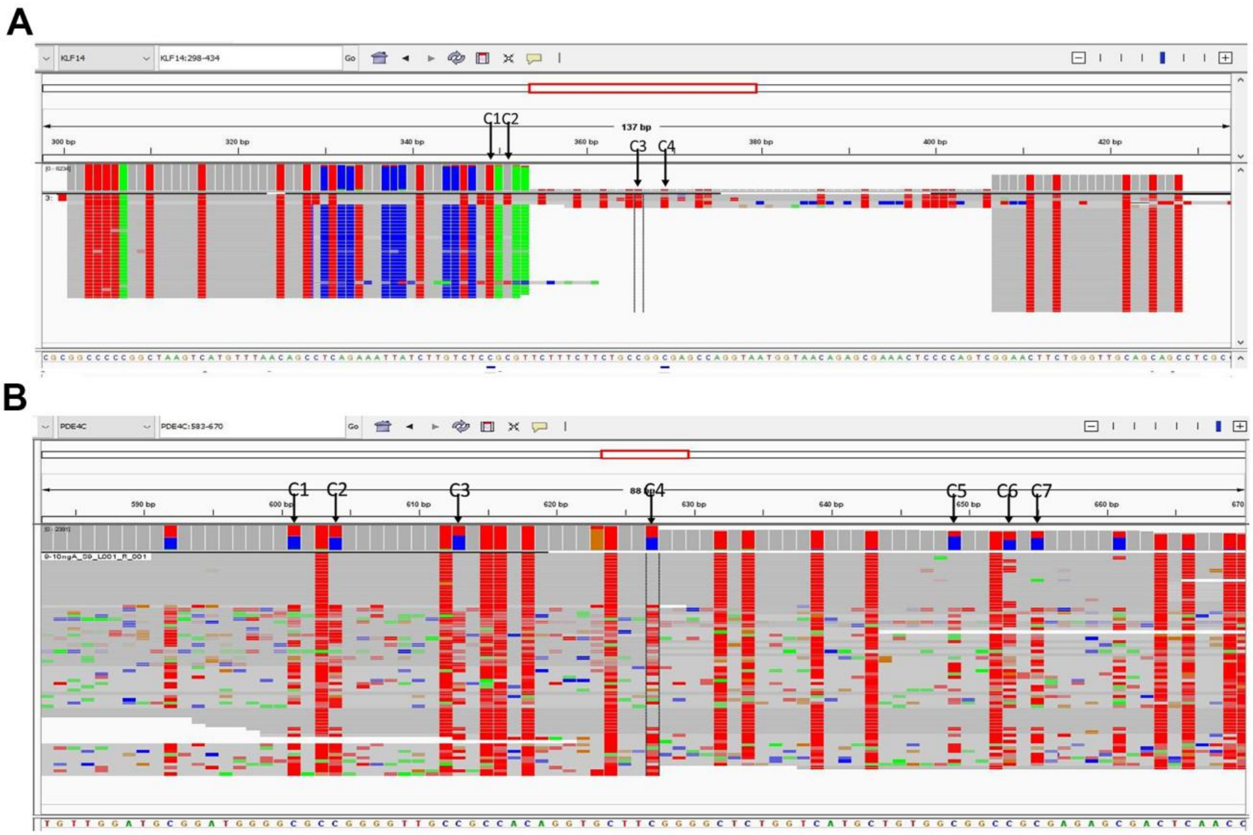

Supplementary Figure 3. (**A**) IGV capture of alignments for NTC-2 *KLF14* amplicon. Alignments are viewed as pairs and shown in squished mode. Target CpG sites are indicated by arrows. (**B**) IGV capture of alignments for the low quantity 10 ng sample. Alignments at *PDE4C* are viewed as pairs and shown in squished mode. Target CpG sites are indicated by arrows.

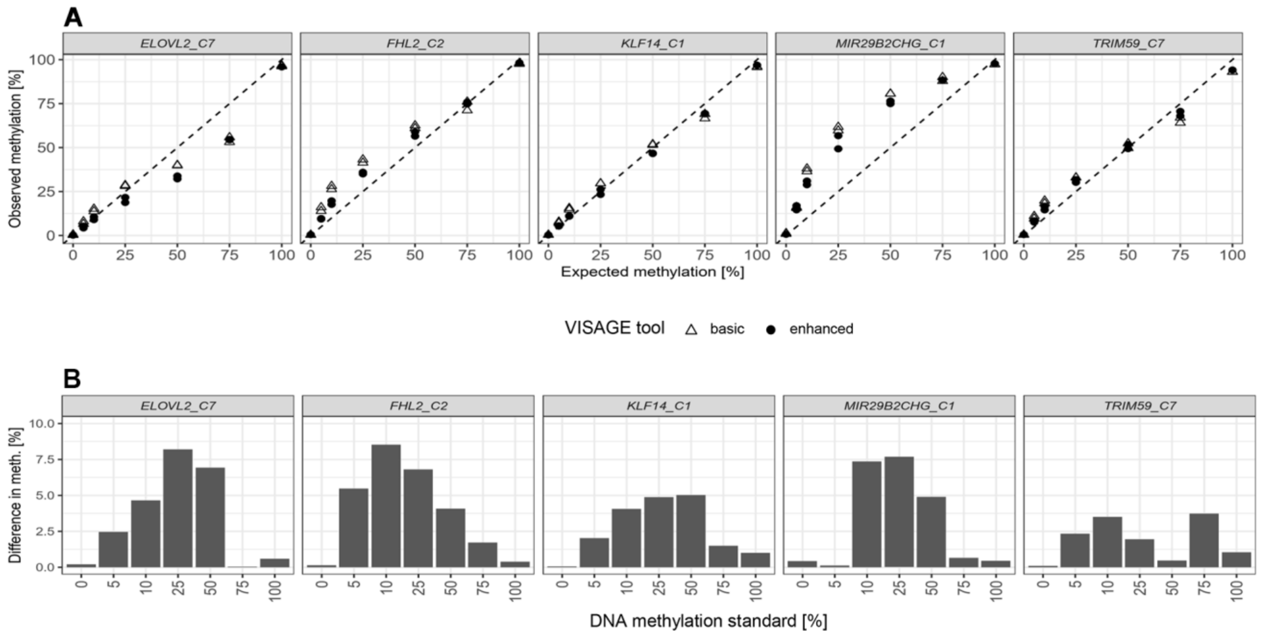

Supplementary Figure 4. (**A**) Methylation values obtained from seven differentially methylated DNA standards processed with the basic or final VISAGE prototype tools. (**B**) Absolute difference between mean quantifications obtained for the two assays.

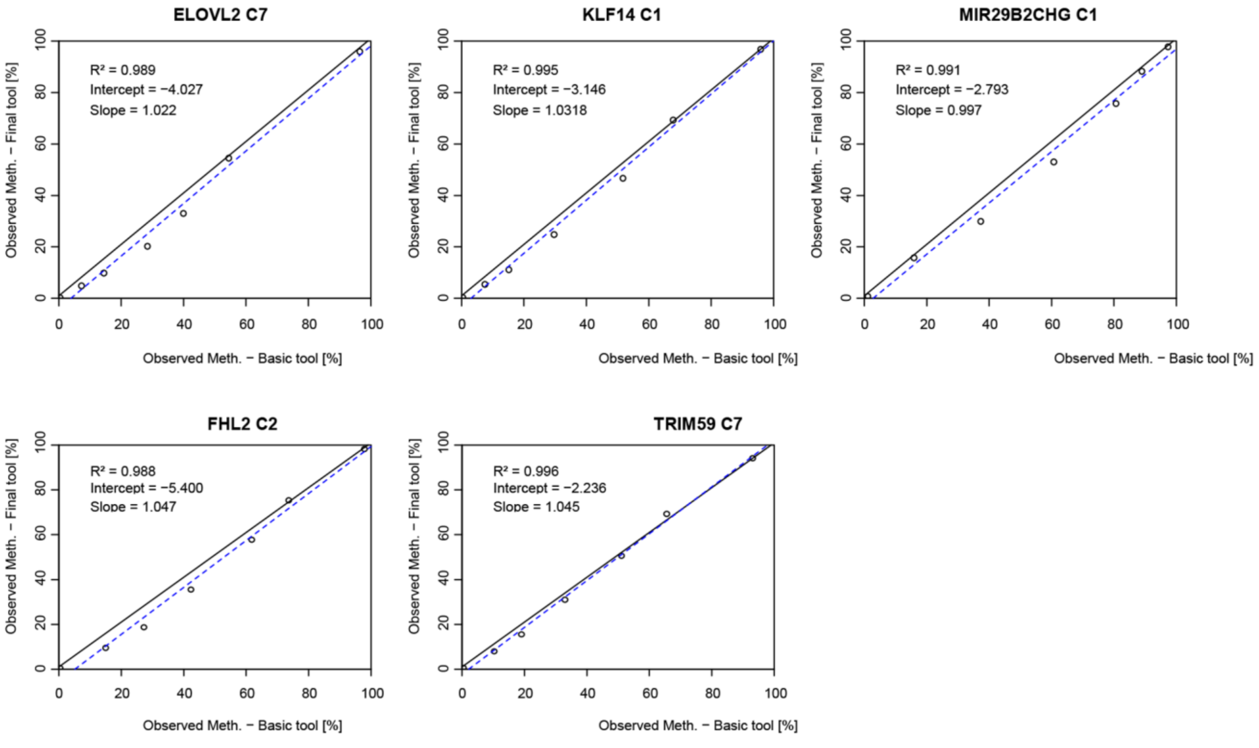

Supplementary Figure 5. Assuming that methylation quantification of the DNA methylation standards using the basic tool or the final tool do not differ significantly, the obtained values should be close to the line of identity (plotted line; intercept = 0, slope = 1). This was tested by comparing the linear regression model based on the experimental data of each marker with the line of identity (regression line is indicated by the dashed, blue line). Results failed to indicate at the 5% Type−I error level a statistically significantly superior performance of the empirical regression model to the line of identity for all five markers (Bonferroni corrected *P*−values: *ELOVL2*_C7: 0.681, *KLF14*_C1: 1.000, *MIR29B2CHG*_C1: 1.000, *FHL2*_C2: 0.756, *TRIM59*_C7: 1.000).

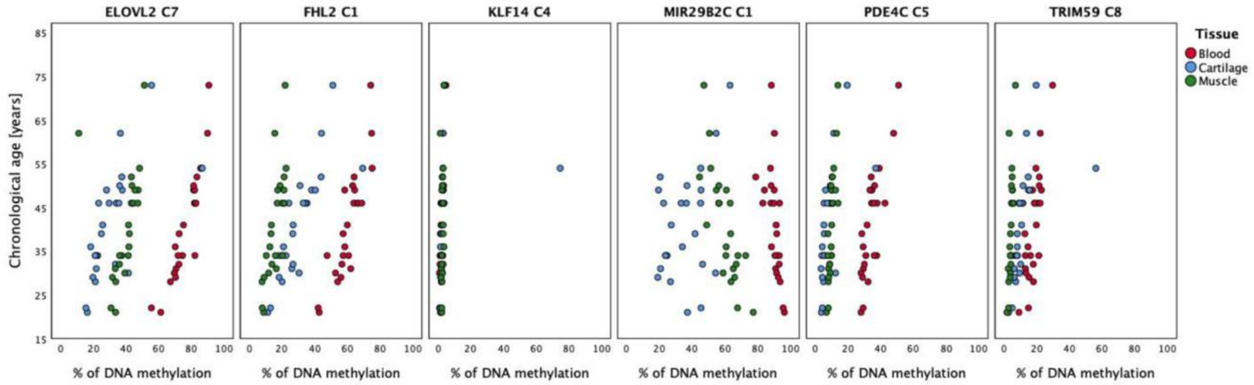

Supplementary Figure 6. Correlation between DNA methylation and chronological age in 3 tissue types collected from 24 deceased individuals.
