# Supplementary material for: Development of the VISAGE enhanced tool and statistical models for epigenetic age estimation in blood, buccal cells and bones
Source: Aging (Albany NY). 2021 Mar 11;13(5):6459–84. doi: 10.18632/aging.202783 (PMC7993733; doi:10.18632/aging.202783)
Supplement: Supplementary Table 2 [file aging-13-202783-s004.pdf]

Supplementary Table 2. Average difference of measured methylation values between duplicates (5% to 75% methylated DNA standards; N = 5) obtained for all CpG sites per marker.

Marker	CpGs (N)	Mean	SD	Median	Minimum	Maximum
ASPA	*1*	1.39	1.59	0.69	0.13	3.81
EDARADD	*2*	1.26	0.78	1.68	0.05	2.21
ELOVL2	*9*	1.23	0.76	1.03	0.02	2.89
FHL2	*10*	1.26	0.82	1.18	0.14	2.79
KLF14	*4*	0.81	0.98	0.36	0.01	2.9
MIR29B2CHG	*3*	3.01	2.45	2.62	0.13	7.46
PDE4C	*7*	4.76	3.69	3.94	0.47	12
TRIM59	*8*	1.74	0.6	1.74	0.56	2.85
overall		*1.93*	*1.23*	*1.33*	*0.81*	*4.76*
