# Supplementary material for: Development of the VISAGE enhanced tool and statistical models for epigenetic age estimation in blood, buccal cells and bones
Source: Aging (Albany NY). 2021 Mar 11;13(5):6459–84. doi: 10.18632/aging.202783 (PMC7993733; doi:10.18632/aging.202783)
Supplement: Supplementary Table 4 [file aging-13-202783-s006.pdf]

Supplementary Table 4. List of sequencing runs for data collecting, used Illumina® MiSeq Kits, library concentration, % PhiX Control and number of pooled samples (including methylation standards 0% and 100%).

Run	Run name	Used kit	Lib. conc. [pM]	% PhiX control	No. samples
1	Swab1	MiSeq Reagent Kit Micro v2 300 cycles	7	1	40
2	Blood1	MiSeq FGx v3 600 cycles	7	5	40
3	Blood2.1	MiSeq FGx v3 600 cycles	9	5	62
4	Blood2.2 *	MiSeq FGx v3 600 cycles	11	5	62
5	Blood3	MiSeq FGx v3 600 cycles	12	5	67
6	Swab2	MiSeq FGx v3 600 cycles	12	5	62
7	Swab3	MiSeq FGx v3 600 cycles	12	5	63
8	Swab+blood*	MiSeq Reagent Kit Nano v2 300 cycles	10	5	8
9	Swab+blood*	MiSeq Reagent Kit Nano v2 300 cycles	10	5	5
10	Tissue	MiSeq Reagent Kit v2 300 cycles	10	5	72
11	Bones1+blood*	MiSeq Reagent Kit v2 300 cycles	10	5	64
12	Bones2	MiSeq FGx v3 600 cycles	12	5	72
13	Bones3+blood*	MiSeq FGx v3 600 cycles	12	5	67

*additional runs for some samples due to missing data.
